# Action Initiation in the Human Dorsal Anterior Cingulate Cortex

**DOI:** 10.1371/journal.pone.0055247

**Published:** 2013-02-27

**Authors:** Lakshminarayan Srinivasan, Wael F. Asaad, Daniel T. Ginat, John T. Gale, Darin D. Dougherty, Ziv M. Williams, Terrence J. Sejnowski, Emad N. Eskandar

**Affiliations:** 1 Neural Signal Processing Laboratory, Department of Radiology, University of California Los Angeles, Los Angeles, California, United States of America; 2 Department of Neurosurgery, Alpert Medical School, Brown University and Rhode Island Hospital, Brown Institute for Brain Science and the Norman Prince Neurosciences Institute, Providence, Rhode Island, United States of America; 3 Department of Radiology, Massachusetts General Hospital, and Harvard Medical School, Boston, Massachusetts, United States of America; 4 Department of Neurosciences and Center for Neurological Restoration, Cleveland Clinic, Cleveland, Ohio, United States of America; 5 Department of Psychiatry, Massachusetts General Hospital, and Harvard Medical School, Boston, Massachusetts, United States of America; 6 Department of Neurosurgery, Massachusetts General Hospital, and Harvard Medical School, Boston, Massachusetts, United States of America; 7 Howard Hughes Medical Institute, The Salk Institute for Biological Studies, La Jolla, California, United States of America; 8 Division of Biological Sciences, University of California San Diego, La Jolla, California, United States of America; French National Centre for Scientific Research, France

## Abstract

The dorsal anterior cingulate cortex (dACC) has previously been implicated in processes that influence action initiation. In humans however, there has been little direct evidence connecting dACC to the temporal onset of actions. We studied reactive behavior in patients undergoing therapeutic bilateral cingulotomy to determine the immediate effects of dACC ablation on action initiation. In a simple reaction task, three patients were instructed to respond to a specific visual cue with the movement of a joystick. Within minutes of dACC ablation, the frequency of false starts increased, where movements occurred prior to presentation of the visual cue. In a decision making task with three separate patients, the ablation effect on action initiation persisted even when action selection was intact. These findings suggest that human dACC influences action initiation, apart from its role in action selection.

## Introduction

Human dorsal anterior cingulate cortex (dACC) includes the cingulate gyrus and cingulate sulcus from the levels of the genu of the corpus callosum anteriorly to the anterior commissure posteriorly. The human dACC has been implicated in various aspects of action selection, including reward-dependent decision making [1], conflict monitoring [2], [3] and representation of error likelihood [4], [5]. A smaller body of work has suggested that human dACC could influence action *initiation* apart from its role in action *selection* [4], [6]–[8]. A fundamental question is whether action selection and action initiation are separable functions of dACC.

Because action initiation and action selection are co-dependent in many tasks, separating these two roles in human dACC has been difficult. This is because initiation is modulated by components of action selection including reward expectancy [9], error likelihood [4], [5], and decision conflict [2], [3]. Resulting correlations between dACC activity and action selection are intertwined with action initiation. Other experiments on dACC have explored simple reaction time tasks where the action is pre-selected, while initiation was randomly cued [4], [6]–[8]. These studies correlated metabolic and electrophysiological activity in dACC with action initiation in the absence of action selection. This raised the possibility that dACC could causally influence action initiation, apart from its effects on action selection.

Early anatomical work on dACC in monkeys established direct connections from neurons in the cingulate sulcus to primary motor cortex and spinal cord, and preliminary efforts were made through fMRI to identify homologous regions in human. In the seminal monkey work, a group of cingulate motor areas (CMA) were defined through cytoarchitectural examination and electrical stimulation studies on the basis of connections to primary motor cortex and the spinal cord [10]–[13]. By definition, the CMA comprise grey matter lining the monkey cingulate sulcus (dorsal and ventral banks) extending anteriorly to the level of the genu of the corpus callosum and posteriorly to roughly the level of the posterior commissure. In the original definition, the CMA are a subset of the dACC, where the CMA exclude the cingulate gyrus [10]. The CMA are anatomically subdivided by the vertical anterior commissure line (VAC). Anterior to the VAC is the rostral CMA (rCMA). Posterior to the VAC are the dorsal CMA (dCMA) and ventral CMA (vCMA) which are located on the dorsal and ventral banks of grey matter that comprise the cingulate sulcus.

**Figure 1:**
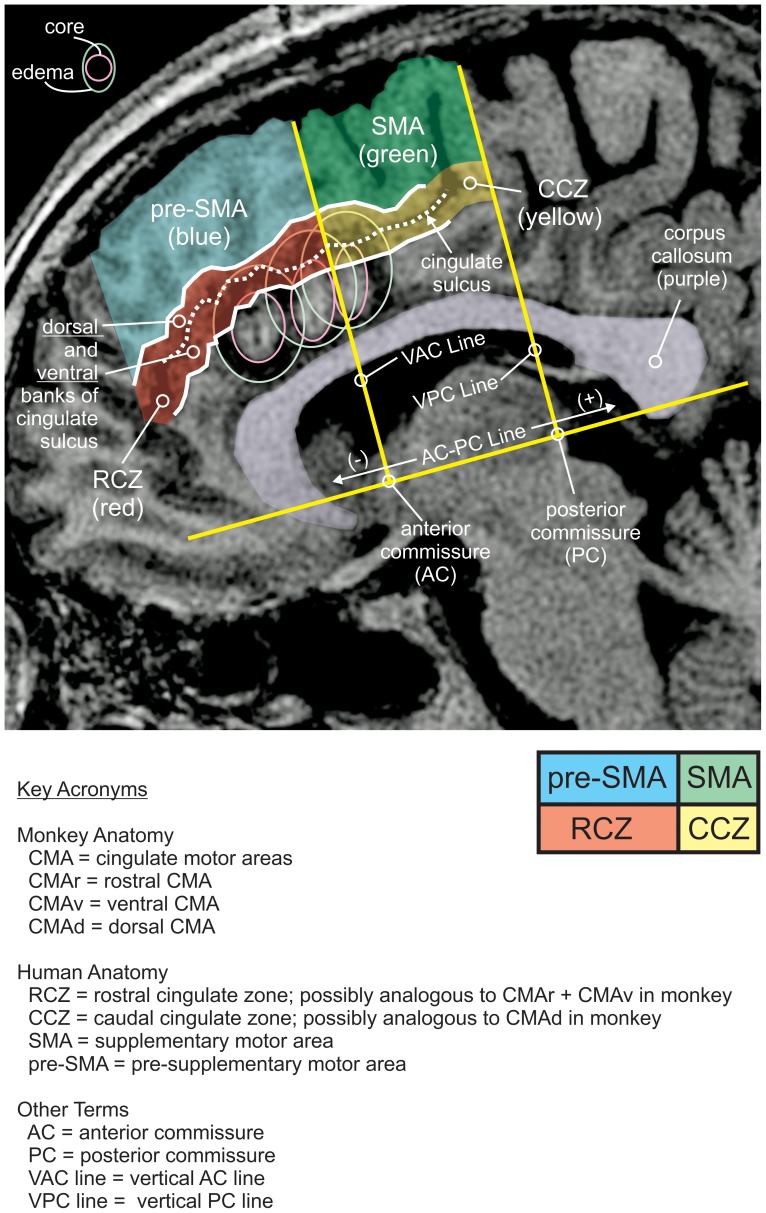
Cingulotomy relative to motor areas of the medial wall as described by Strick and colleagues [12]–[14]. Parasagittal postoperative T1 weighted MPRAGE sequence demonstrates three neighboring foci of radiofrequency (RF) ablation with surrounding edema in the dorsal anterior cingulate cortex (dACC). Three additional stereotactically guided RF ablation foci are located in the contralateral dACC. The patient imaged in this high resolution sequence was more recent to S1–S6. Imaging was performed in the immediate postoperative period, within hours to days following ablation.

The homology between monkey and human cingulate motor areas is widely employed, but this relationship is largely based on resemblance between PET and fMRI activation patterns in human and divisions of the medial frontal cortex in monkey [12], [13]. As noted by the originators of this homology, the sulci and gyri of the human medial frontal cortex are highly variable, so that rigid landmarks that represent cingulate motor areas based on these surface features are not asserted with certainty [13]. Related work by another group provides helpful anatomical labeling on human MRI images [14]. In this framework, the grey matter along the cingulate sulcus is described as the cingulate zones (CZ), depicted in Figure 1. Whether the CZ additionally include the cingulate gyrus is ambiguous. Anterior to the VAC line is the rostral CZ (RCZ). Posterior to the VAC line is the caudal CZ (CCZ). The human RCZ may be comparable to monkey rCMA and vCMA. The human CCZ may be comparable to monkey dCMA. In retrospective analyses of activation patterns during various motor and decision-making tasks in humans via positron emission tomography (PET) [12] and fMRI [13], Strick and colleagues developed a hypothesis that CCZ is involved in simple motor tasks, while RCZ is more involved in complex motor tasks, including conflict monitoring anteriorly (RCZa) and response selection posteriorly (RCZp).

In non-human primate, dACC has been linked to reward-dependent decision making [15]–[18] and the CMA subdivision of dACC has been studied with relation to motor control, although its specific role in motor control is not yet completely clear. To elaborate on this point, we briefly survey the existing non-human primate literature on the CMA and motor control. Intracortical microstimulation of the CMA demonstrated measurable muscle response [11]. These areas exhibit pre-movement and peri-movement neural activity with varying degrees of directionality tuning [19]–[22]. Spiking activity in rCMA is correlated with motor learning [23]. Although these non-human primate studies implicate dACC in some element of motor control, they do not provide substantial additional specificity. In related work, recordings from the dorsal bank of the monkey anterior cingulate sulcus found no correlation between reaction time, movement time, and spiking rates during a decision-making task that required eye and arm movements [17]. In general, these electrophysiology studies are also limited by a correlation-causality confound.

Some work in rat dorsomedial prefrontal cortex (dmPFC) has attempted to provide causal statements about movement initiation, although the homology between dmPFC and human dACC is further removed and controversial [24]. In this work, reversible inactivation of dmPFC with muscimol caused increased premature movement (false starts) in a reaction time task [25]. Electrophysiological recording in primary motor cortex (MI) combined with dmPFC inactivation also demonstrated a delay-period-specific interaction between dmPFC and primary motor cortex [25]. Although these experiments causally relate dmPFC to motor control, they do not concurrently examine action initiation and action selection. Moreover, these results do not extend immediately to the structure and function of human dACC.

Recently, several groups have used human cingulotomy, undertaken for purely therapeutic indications, as an opportunity to study the function of the human dACC [1], [26]–[31]. Cingulotomy involves the bilateral ablation of dACC (Figure 1A). This treatment is performed for intractable obsessive-compulsive disorder (OCD), depression and chronic pain [32]. Only a handful of cingulotomies are performed each year; the number of patients included in this paper represent a significant fraction of those patients [32]. Previously, we studied patients undergoing this procedure to establish a causal link between human dACC and the processes that control action selection [1]. We also previously showed that in decision making, dACC causally modulates reaction times in relation to expected cognitive demand [33]. In this study, we examined the performance of patients undergoing cingulotomy on either a simple reaction time task with a pre-selected action or a reward-dependent decision-making task that required action selection and action initiation. We quantified performance in action initiation and action selection based on rates of false starts and percentages of incorrect decisions respectively. Measurements were taken within minutes before and after bilateral dACC ablation in the intraoperative setting.

## Materials and Methods

### Patient Selection and Surgery

Patients were enrolled in this study through informed written consent under a protocol approved by the Institutional Review Board and the cingulotomy assessment committee at Massachusetts General Hospital. The decision to offer surgery bore no relation to the current study, and adhered to the same evaluative and ethical guidelines used for all prior cingulotomy patients. At all time points, patients had the understanding that their participation bore no relation to the surgical outcome, and that they could withdraw from the study at any time.

**Figure 2:**
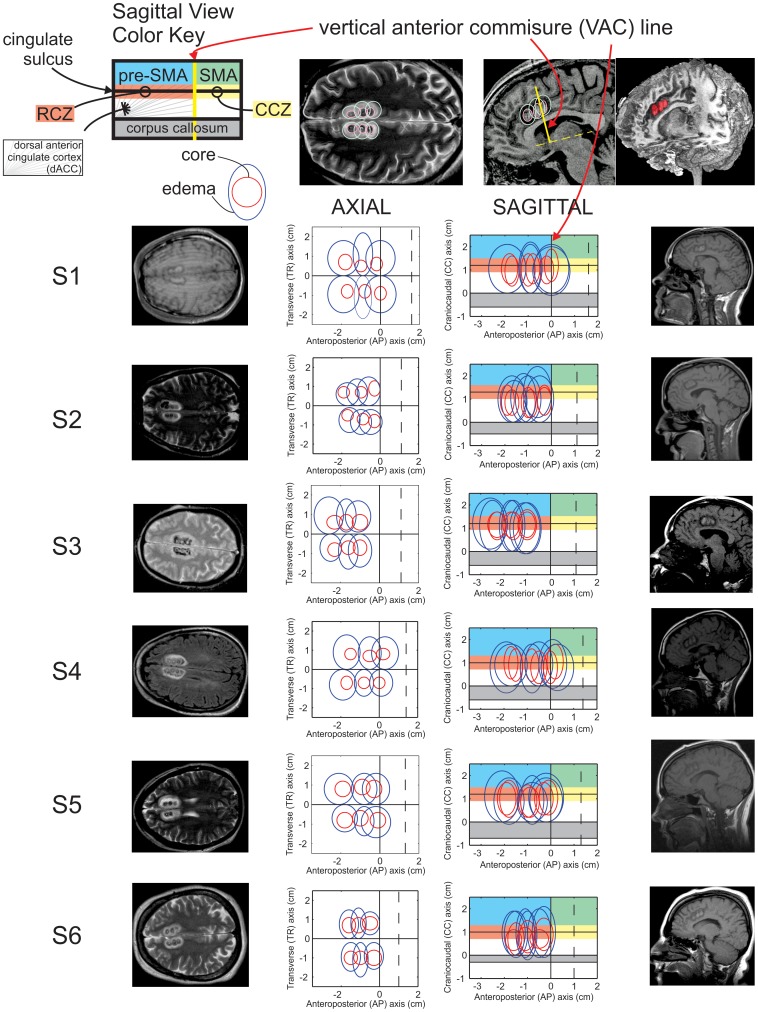
RF ablation zones for all subjects in axial and sagittal projections. Core ablation foci (red) and surrounding edema (blue) are approximated as concentric ovals. Anteroposterior (AP) measurements are made relative to the VAC line. Transverse (TR) measurements are made relative to the interhemispheric fissure. Craniocaudal (CC) measurements are made relative to the superior margin of the corpus callosum. The extent of motor areas, shaded in color, is only approximate. The vertical dotted line (sagittal) indicates the paracentral sulcus. Red foci in the 3D reconstruction (top right) include both core and edema. Imaging was performed in the immediate postoperative period, within hours to days following ablation.

**Figure 3:**
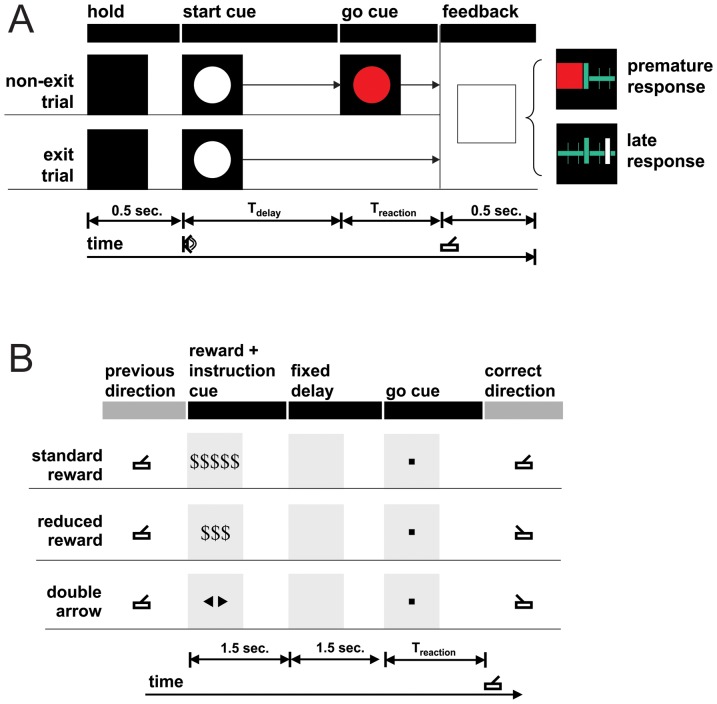
Tasks. (**A**) The simple reaction time task presents trials with (non-exit) and without (exit) go cues. The delay period (T_delay_) and trial type are randomized to discourage anticipatory strategy. A feedback period indicates to the patient whether their movement preceded the go cue (premature response), or followed the go cue (late response). The thick green tick mark indicates the time of the go cue. The thin green tick marks indicate 10 millisecond intervals on a time axis (medium horizontal green line). The large red square is an icon representing premature response. The white tick mark indicates the reaction time relative to the go cue, provided with late responses. (**B**) The reward-dependent decision making task includes components of action selection and action initiation. The reward+instruction cue indicates the reward schedule and prompts the user to decide left versus right joystick movement. The go cue prompts the user to initiate movement. The pictures under “reward+instruction cue” and “go cue” are the exact icons presented to the patient. See article text for full description of the two tasks.

Six patients were studied before and after bilateral radiofrequency ablation of dACC (also called cingulotomy) as a last-resort surgical treatment, illustrated in Figure 1 with a different patient (not S1–S6) using a high-resolution T1-weighted structural MRI scan (MPRAGE). Each patient was a unique individual (Figure 2). No patients had a history of prior brain surgery. Detailed clinical selection criteria and surgical procedure are described elsewhere [34]. Each patient participated in only one of two tasks, with three patients in the simple reaction time task, and three different patients in the reward-dependent decision making task (Figure 3). Patients that were enrolled in the reaction time task (S1–S3) met DSM-IV-R criteria for axis I diagnosis of OCD. Patients that were enrolled in the reward-dependent decision making task (S4–S6) included one patient diagnosed with OCD (S4) and two patients meeting DSM-IV-R criteria for major depression (S5, S6).

### Data Collection Periods

**Figure 4:**
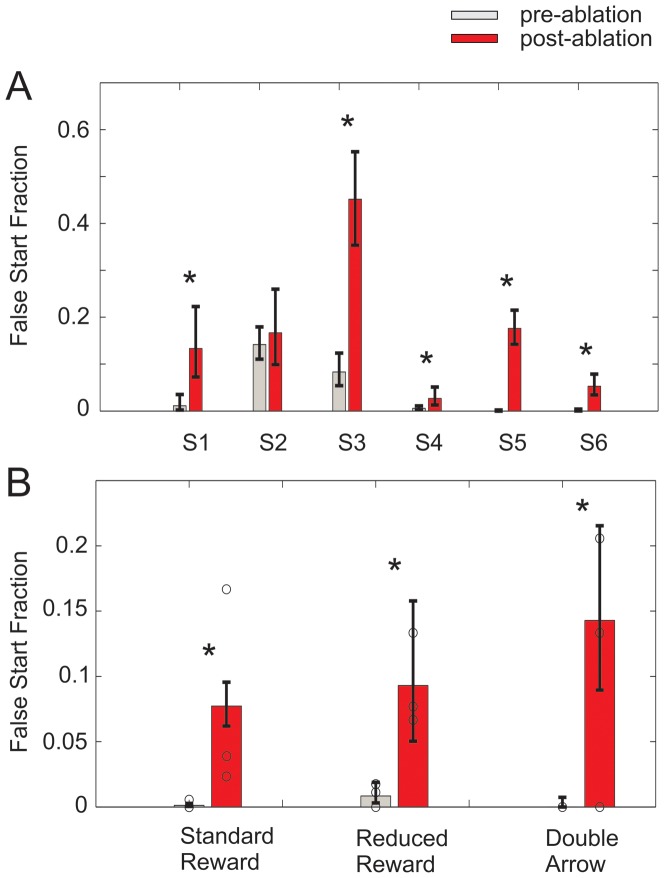
Fraction of premature responses before (gray) and after (red) bilateral dACC ablation. (A) False start fractions in six patients for the simple reaction time task (S1–S3) and the reward-dependent decision making task (S4–S6). (B) False start fractions in the three conditions of the reward-dependent decision making task, grouping across patients (S4–S6). Circles mark individual false start fractions for S4–S6. In both panels, asterisks indicate p<0.05, and error bars indicate 66% confidence intervals, analogous to standard error of the mean (standard error bars). For bars in panel A, proceeding from left to right, N = 90, 30, 120, 30, 84, 31, 533, 112, 816, 125, 805, 133. For bars in panel B, proceeding from left to right, N = 1676, 285, 239, 43, 239, 4.

Patients were trained on the tasks prior to surgery. Pre-ablation data was collected while the patient was situated on the operating table *after* two small bilateral burr-holes were made to introduce the ablation electrode. Post-ablation data was collected under identical intra-operative conditions, within 5 minutes after completing bilateral dACC ablation and approximately 30 minutes after the last pre-ablation trial. The task was run in blocks of 30 or 40 trials. Extra-operative and training trials were excluded from analysis. Total trial numbers are listed in Figure 4 captions.

### Simple Reaction Time Task

The simple reaction time task (Figure 3A) consisted of exit trials and non-exit trials. All trials began with a minimum 0.5 second hold period which required the joystick to be centered, followed by a start cue, indicated by the appearance of a white circle on a dark screen. In exit trials, this white circle was extinguished after 2 seconds, and a feedback screen was presented. In nonexit trials, the white circle turned red after a random interval of time (T_delay_), and the feedback screen was presented after a movement was detected, or 3 seconds from the go cue, whichever came first. Patients were instructed to pull the joystick, initiating movement as rapidly as possible following appearance of the red circle, but not prematurely. This instruction to the patients was identical for all trial types, including exit and non-exit trials. Patients were not explicitly cued whether trials were exit or non-exit type. The feedback screen provided a pictorial representation of premature response or non-premature reaction time. The onset of the white circle was accompanied by a brief acoustic tone. To choose trial type and T_delay_, a number T_exp_ was drawn from an exponential distribution. For T_exp_>1, an exit trial was presented. For T_exp_<1, a non-exit trial was presented with T_delay_ = 1+T_exp_. The exponential distribution parameter was chosen such that 40 percent of trials were exit trials. Reaction tasks commonly elicit anticipatory rather than reactive behavior because go cues are predictable [25], [35]. Our task rigorously extends the “non-aging foreperiod” [36] to guarantee a constant instantaneous probability of go-cue appearance that keeps cues more unpredictable.

### Reward-Dependent Decision Making Task

The reward-dependent decision making task (Figure 3B) consisted of three trial types: standard reward, reduced reward, and double arrow. In each trial type, the patient moved a joystick left or right based on the presented cues. The patient was presented with two cues. First, a reward+instruction cue indicated to the patient the amount of reward and the specific direction needed to move in this trial. Second, a go cue indicated the patient should initiate their chosen joystick movement. The standard reward icon instructed a movement in the same direction as the previous trial, and the reduced reward or double arrow icons instructed a movement in the opposite direction as the previous trial. The standard reward and double arrow icons represented an equal amount of reward, and the reduced reward icon represented a decreased reward. Accordingly, trial types varied in reward schedule and difficulty. Action selection data from the three patients (S4–S6) have been reported previously along with aggregate raw reaction times grouped across all patients and conditions [1].

### Patient Training

Patients were instructed and trained in their respective tasks for a cumulative time of less than 30 minutes in a day preceding surgery, or in the minutes preceding entry into the operating room. There was no evidence for learning, including the more complex reward-dependent decision making task as reported previously [1].

### Data Acquisition and Analysis

Behavioral control software was written in Matlab with the Data Acquisition Toolbox and Space Variant Imaging Toolbox (http://svi.cps.utexas.edu/software.shtml). Visual stimulus times were detected with a photoresistor, and movement onset time was recorded for feedback as the first detectable displacement from origin based on a simple threshold. Joystick displacements and photoresistor outputs were separately recorded with a data acquisition system (Power-1400, Cambridge Electronic Design, Cambridge, UK) for off-line detection of stimulus and movement onset times based on simple thresholding in MATLAB. Incidence of false start fractions was estimated with the Clopper-Pearson interval. Significance testing of differences in false start fractions between pre- and post-lesion sessions was performed using a two-tailed Pearson Chi-square test at p<0.05.

## Results

### Anatomical Analysis

As shown in a representative high resolution (MPRAGE) T1-weighted MRI scan of a patient different from S1–S6 (Figure 1), each hemisphere received three ablations along the dACC. MRI images were obtained in the immediate postoperative period (hours to days). These images show that each ablation site includes a core and a surrounding zone of edema. To first approximation, the ablation core progresses to a CSF-filled cavity over months while the zone of edema resolves. Although creation of the core zone is likely tightly coupled in time to the surgical ablation, the speed of onset of peripheral edema is not certain. Not apparent in this image are peri-millimeter tracks from the vertex to the ablation zone that result from traversal of the stereotactically placed radiofrequency ablation tip.

We examined the ablation extent with relation to the previously defined discrete motor areas of the human medial frontal cortex [12]–[14] as discussed above. Figure 1 delineates the locations of the supplementary motor area (SMA), pre-SMA, caudal cingulate zone (CCZ), and rostral cingulate zone (RCZ) with relation to the vertical anterior commissure (VAC) line and cingulate sulcus. We measured the ablation foci in patients S1–S6 with relation to these various medial wall motor cortical areas (Figure 2). These measurements confirm that the extent of ablation primarily involves the RCZ across all patients. There is minimal or no extension into the corpus callosum. The extent of involvement of parenchyma superior to the cingulate is variable. Color coding in Figure 2 designates approximate boundaries of motor areas that are proposed in homology to monkey anatomy by Strick and colleagues [12]–[14]. Per this approximation, portions of the SMA are involved by one ablation core in S1and S4, and edema in S2 and S5. The SMA is relatively spared in S2, S3 and S6, where superior extension primarily involves the pre-SMA. Of incidental note, S2 demonstrates ablation cores that involve the dorsal bank of the cingulate sulcus to a lesser extent than the other subjects. This may relate to the lack of increased false starts in Figure 4A (to be discussed below), although this possibility is speculative in the absence of a larger study group or an anatomical equivalent of a dose-response curve.

### Behavioral Analysis

We first examined the fraction of movements generated prior to the go cue. Figure 4A plots the false start fraction immediately before (gray) and after (red) bilateral dACC ablation for six patients. Recall that S1–S3 participated in the reaction time task, and S4–S6 participated in the reward-dependent decision making task. Across patients with significant (p<0.05) change in reaction time (S1, S3, S4, S5, S6), the false start fraction increased variably between 3 and 37 percent following ablation. For patient S2, a 2 percent increase in false start fraction following ablation did not meet significance. This was likely due to elevated pre-ablation false start rates versus other patients; ablation measurements (Figure 2) did not suggest a grossly different ablation pattern in S2, as ablation cores were essentially contained within RCZ. In summary, the effect of ablation on false start rates was sufficiently pronounced that it was detected independently in nearly every patient. Moreover, the effects occurred despite explicit instructions against premature movements and prominent visual feedback on performance.

We also analyzed false start fractions in comparison to action selection errors in the reward-dependent decision making task (S4–S6). We previously reported for these patients that bilateral dACC ablation profoundly increased action selection errors in the reduced reward and double arrow conditions, but not in the standard reward condition [1]. Grouping across S4–S6, action selection error rates were 62+/−8 percent and 28+/−9 percent in the reduced reward and double arrow tasks respectively, but were negligible in the standard reward condition [1].

In Figure 4B, we grouped false start events from S4–S6 along these same three conditions to compare action selection errors against false start rates. The resulting false start analysis (Figure 4B) demonstrated increases in false start fractions in each of the three conditions of the reward-dependent decision making task (p<0.05). The confidence intervals in this grouping were wider than in the Figure 4A analysis. To examine the source of this variance, we plotted individual patient false start fractions (circles). The vertical spread in the locations of the circles reflects cross-patient variability that did not contribute to confidence intervals in Figure 4A where data had been separated by patient. Notably, hypothesis testing for individual task conditions separated by patient showed a consistent ablation effect in S5 and S6 across task conditions, but not in patient S4. However, when condition types were grouped for S4 (Figure 4A), the ablation effect on false starts achieved significance. This suggested that for S4 in particular, the ablation effect on false starts was relatively small. Separating the total set of trials performed on this patient by task condition left insufficient trials to detect the ablation effect.

In order to determine the extent of statistical dependence between choice errors and false starts, we asked what percent of false start movements also registered correct choice answers, relative to the overall percentage of false start movements. In technical terms, recall that statistical independence between two random variables x and y is equivalent to p(x) = p(x|y). In reduced reward and double arrow trials following dACC ablation, choice errors were under-represented in false start trials, inconsistent with statistical independence in reduced reward and double arrow conditions. Specifically, only 16+/−14 percent of false start trials resulted in choice error, despite an overall choice error of 46+/−2 percent in these trials (p<0.05). For standard reward trials, the rate of choice error in false start trials was indistinguishable (p<0.05) from the overall rate of choice error (nearly 0 percent), consistent with statistical independence in standard reward conditions. There, only 1 trial out of 42 trials resulted in choice error, which was also a false start trial.

Finally, we examined the effect of ablation on late responses in the reaction time task. A late response was defined as a response where no movement was registered when a go cue was issued. Intuitively, the percentage of late response is a measure of task engagement. In S1 and S2, no late responses were registered in any testing. In subject S3, late response percentages were also not significantly changed following ablation. In this subject, percentage late response measured 7% pre-ablation (95% confidence intervals ranging 2 to 20%) and 15% post-ablation (95% confidence intervals ranging 3 to 46%) post-ablation.”

## Discussion

In navigating daily life, we are frequently called upon to make choices (action selection), and to act upon those choices (action initiation). Previous research has implicated the dACC in action selection, with other correlative studies suggesting it may also be involved in action initiation. Our results provide evidence that dACC is causally involved in action initiation, separate from its involvement in action selection. We demonstrated that anatomically and temporally focused dACC ablation immediately disrupted action initiation even when action selection is intact or when actions are pre-selected. Below, we discuss issues of correlation versus causality, and address possible confounds in interpretation. We conclude with a discussion of our results in the context of the various prevalent models of dACC function.

### Correlation versus Causality

Establishing causality between human dACC and elements of behavior has been challenging for a variety of reasons. Imaging and electrophysiology studies are fundamentally correlative. Transcranial magnetic stimulation (TMS) has been useful in establishing causal roles for more superficial cortical structures [37], but dACC lies several centimeters deep to the scalp surface. Lesions caused by disease are anatomically heterogeneous, usually unilateral, and likely associated with some degree of functional reorganization over time. Pathological lesions often concomitantly involve other brain areas [38]. Even seemingly focal lesions such as from stroke or tumor resection are rarely confined to discrete, functionally-restricted subdivisions of the brain. Functional reorganization in the months following these sorts of lesions also cannot be excluded as a variable in retrospective studies involving stroke [39] and tumor [40]. Hence the ability to detect causal relationships from these lesions [41]–[43] is severely limited by anatomical and temporal variability between patients.

In comparison, cingulotomy provides several comparative advantages to pathological lesion studies. The ablation is focal, surgically controlled in position, extent, temporal onset, and mechanism (thermocoagulation), and readily visualized by MRI. The effects of dACC ablation can be studied intra-operatively, immediately preceding and following ablation. This minimizes potential variability due to cortical re-organization, mass effect from post-ablation edema, or behavioral adaptation.

### Possible Confounds in Interpretation

Could increased premature responses following ablation be entirely explained by dACC's causal role in reward-dependent decision making [1]? Alternatively, could changes in arousal or motivation [42], or global disinhibition explain increased premature responses? Could the demands of task switching in the reward-dependent decision making conditions trivially decrease post-ablation performance? False start fractions in the various conditions of the reward-dependent decision making task addressed these potentially confounding interpretations. Although levels of reward and task difficulty varied across the standard reward, reduced reward, and double arrow tasks, dACC ablation resulted in increased false start fractions in all of the three tasks (Figure 4B). In contrast, dACC ablation increased action selection errors in only the reduced reward and double arrow tasks, where action selection was intact in the standard reward task [1]. This key difference helps to resolve the aforementioned confounding interpretations.

The experimental design helps to address several other potential confounds. It is unlikely that the action initiation deficit is a simple placebo effect of the ablation, as patients were not told to expect any action initiation deficit and the action initiation deficit did not relate to the expected therapeutic benefits for OCD, depression, or bipolar affective disorder. The effect is unlikely to be specific to a particular experimental paradigm because the action initiation effect persisted across two entirely separate tasks. Finally, the ablation effect on action initiation is unlikely to be attributable to operating room conditions or neurosurgery. This is because our experiment allowed each patient to serve as their own internal control where pre- and post-ablation data was collected under the same intra-operative conditions. Additionally, the pre-ablation data were collected following placement of a stereotactic frame and the start of surgery, so that effects of these neurosurgical interventions would have been present in the pre-ablation data. The post-ablation trials occurred within 5 minutes of the ablation procedure, temporally associating the observed deficits with the ablation event itself.

Because this experiment represents a surgically invasive human study, performing the lesion experiment on healthy volunteers is not an option. As a result, we cannot be certain that the effects of dACC ablation on action initiation necessarily generalize to the healthy brain. However, we can be reassured that the ablation effects were not specific to any particular disorder. Specifically, two of these patients received dACC ablation for otherwise refractory major depression or bipolar affective disorder while the remainder had OCD. Moreover, as discussed above, the surgical ablation procedure mitigated concerns raised by retrospective stroke or tumor studies, such as effects of cortical plasticity or paraneoplastic phenomena. Finally, prior work that implicated dACC in action initiation used healthy volunteers [4], [6]–[8].

Our analysis confirms dACC ablation results in action timing deficits even when decision making is intact (standard reward condition of the reward-dependent decision making task) or when decision making is entirely removed from a task (simple reaction time task). Consequently, deficits in action timing are not simply an epiphenomenon of dACC's role in decision making. Paradoxically, when choice errors and false starts coexist (reduced reward and double arrow conditions), choice error is decreased in false start trials relative to the overall choice performance. This is contrary to what might be expected from noisy integration in an integrate-to-threshold model, where false starts would be enriched with choice errors. Instead, under these conditions, dACC ablation may actually allow choice certainty to potentiate false starts.

With regards to reaction times, our prior work on reward-dependent decision making showed that S4–S6 together demonstrated no significant difference in reaction times between correct and incorrect decisions [1]. On a single subject basis, reaction times following ablation showed heterogeneity, with some showing significant decrease (S1, S4, S5, S6), and others showing significant increase (S2, S3) at the two-tailed p<0.05 significance level. The literature also equivocates on the result of ablation on reaction times. Slowed reaction times were observed in one study of ocular saccades with two patients following small strokes that affected RCZ (just anterior to the VAC line) in comparison with normal controls [44]. In another study of dorsal cingulate sulcus, in nonhuman primate, contralateral reversible muscimol-based inactivation of neurons resulted in decreased reaction times and movement times as well as disruption of decision-making [16]. Our recent work on human dACC in ongoing behavioral adaptation might reconcile these varied results [33]. This work suggests that dACC modulates reaction times in relation to expected cognitive load on a trial-by-trial basis (rather than indiscriminately elevating or decreasing reaction times), so that loss of this function might increase or decrease reaction times depending on the nature of the task.

### Relationship to Prevalent Theories of dACC Function

#### Medial Frontal Cortex in Response Inhibition

One prevalent theory suggests that areas of medial frontal cortex are variously involved in response inhibition, arising from early studies of anatomically non-specific medial frontal lesions in humans during go/no-go and related tasks [45]–[48]. Our present study clarifies the contribution of dACC lesions in particular as a specific focus within medial frontal cortex. While response inhibition more generally encompasses action timing and action selection, our results clarify that dACC lesions can result in disinhibition of action initiation when action selection is intact (reward-dependent decision making task), and this disinhibition can be seen even when no action selection is required (reactive task).

Is it possible that damage to SMA could explain the ablation effects? Prior work in monkey had investigated spiking neural activity in SMA versus CMAd and CMAv in a classical delayed instructed center-out reaching task [22]. In this work, SMA was more closely related to pre-movement neural activity than CMAd or CMAv. All three regions were modulated during visual cue onset and during arm movement. This correlative study suggested the possibility that SMA could be preferentially involved in movement onset. Our analysis does not invalidate this possibility. However, SMA (as defined by Strick and colleagues, Figure 1) appears to have been essentially spared in S2, S3 and S6. With ablations primarily centered on RCZ, a minimal involvement of SMA is not a likely explanation. Nevertheless, fMRI may be helpful in the future to delineate a functional (rather than purely anatomical) definition of SMA prior to ablation in clarifying this point.

#### Medial Frontal Cortex in Voluntary Actions

A complementary theory based on primate and human studies states that medial frontal cortex participates selectively in voluntary rather than reflexive actions [49], including supplementary motor area (SMA), supplementary eye field (SEF), and pre-SMA [14], [50]–[55], and cingulate motor areas [56]. The terms “voluntary” and “reflexive” are imprecise, but one defining criterion states that under voluntary behavior, an appropriate action is not uniquely and unambiguously specified by a given set of stimulus conditions [49]. This definition more broadly circumscribes theories that relate to dACC in decision making. With regards to this line of thought, our results unambiguously demonstrate that dACC can disrupt the ability to reliably generate reflexive behavior, such as in the reactive task where there is no component of voluntary behavior, at least by the definition for voluntary behavior given above. Although our data contradicts this theory, it does not contradict the body of evidence that supported this theory, because this literature generally has relied on fundamentally different lesions, frequently old, with heterogeneous locations and significant global pathology such as stroke and cancer. Related to this theory is the notion that voluntary behavior requires the inhibition of reflexive actions that are driven by external stimuli, which is disrupted in two patients with prior pre-SMA and SEF lesions respectively [57]. This related notion offers limited insight into the behavior observed in our reactive task. In contrast to the priming stimulus used in [57] to provoke incorrect responses, the false starts generated by dACC lesions in our reactive task are unprovoked, occurring the complete absence of go cues.

#### Accumulator Models of Reaction Time

Accumulator models describe reaction time as a consequence of the integration of a noisy signal, with actions initiated when this integration crosses a threshold. These models are mathematically compact. However, accumulator models are black box, i.e. they are not biophysically detailed. These models are not specifically related to anatomy of dACC or any other brain region. This is problematic because it limits the model's practical utility in predicting the effects of lesions immediately beyond the vicinity of the area studied. At their core, these models are parametric descriptions of the data for a specific experimental setting, with limited predictive power beyond the conditions of the experiment.

A counter argument in support of accumulator models is that by mapping changes in reaction time distributions onto changes in parameters of an accumulator model, results can be interpreted in terms of this model's language. For example, we might say that the effect of dACC lesions was to lower the decision threshold, or to increase the noisiness of integration. Even if the equivalent effect on accumulator model parameters was relevant in some context, pre-ablation false start rates in this experiment were nearly zero in 4 out of 5 patients that demonstrated the effect (see Figure 4A). As a result, fitting accumulator model parameters to describe nearly faultless behavior is ill posed, because any number of large thresholds or low noise parameter values could account for nearly absent false starts. Following the lesion, total trial numbers are limited by the intra-operative nature of the study, again resulting in an underpowered parameter fitting exercise in choosing between increases in accumulator noise or decreases in threshold. Accordingly, this modeling strategy was not pursued.

#### Conflict Monitoring Signal from dACC as Input to Subthalamic Nucleus

The theory by Frank, et. al in [58] describes subthalamic nucleus (STN) as a brake on movement onset in response to a “conflict monitoring signal” provided by anterior cingulate cortex. A decision-making trial with two nearly equivalent options is called “high conflict,” while a decision-making trial with two disparate options is called “low conflict.” This prior work showed that deep brain stimulation (DBS) of the subthalamic nucleus (STN) resulted in faster reaction times for high conflict trials, where those trials would ordinarily elicit slower reaction times in comparison with low conflict trials. The overall percentage of incorrect (suboptimal in terms of expected reward) choices was unchanged by DBS. The authors of [58] present these results in support for a model of action selection and initiation where the STN regulates action initiation “by effectively raising decision thresholds in the face of conflict.”

Interestingly, the authors posit that STN derives the level of conflict from cingulate and neighboring areas, supported by anatomical connectivity and incorporation of the separate finding that cingulate fMRI activation correlates with conflict level [59]. Our results show that disruption of activity in cingulate is capable of directly inducing premature action initiation, even when STN is intact. From the perspective of the model in [58], perhaps that absence of dACC input signals low conflict, lowering the decision threshold via STN, resulting in faster responses. However, it is difficult for our results to completely support models such as [58], or the accumulator model, because such models tightly couple action timing and action selection by invoking a decision threshold that also initiates action, while our data shows that these functions can be separately disrupted by combining dACC ablation with appropriate reward schedules, such as with our analysis of the reward-dependent decision making task.

### Non-Exclusivity of dACC to Action Initiation

Both previous studies and our current results suggest that dACC is not the unique, exclusive determinant of action initiation. Ablation studies of comparable spatial and temporal precision are not readily available for other regions of human frontal cortex. However, retrospective studies of heterogeneous frontal lesions have demonstrated response inhibition as being affected by various areas of medial and lateral frontal cortices [45], [60], [61]. The dACC is functionally interconnected with cortical areas including the medial temporal lobe, the posterior cingulate cortex, the adjacent precuneus and the medial, lateral and inferior parietal cortex, and corresponding areas of basal ganglia [62]. Although premature responses in our own data increased following ablation, the false start fraction typically remained under 25 percent rather than jumping to nearly 100 percent as might be expected if dACC was the exclusive determinant of action initiation. Together, these results suggest that other brain regions are likely capable of contributing to action initiation, along with the dACC.
